# 基于阳离子型金属有机骨架混合基质膜的分散固相萃取-超高效液相色谱-串联质谱法测定水中7种苯氧羧酸类除草剂

**DOI:** 10.3724/SP.J.1123.2021.01006

**Published:** 2021-08-08

**Authors:** Xuefeng JI, Shuang LI, Gege WU, Lin ZHAO, Jiping MA

**Affiliations:** 1.青岛理工大学环境与市政工程学院, 山东 青岛 266033; 1. School of Environmental and Municipal Engineering, Qingdao University of Technology, Qingdao 266033, China; 2.泰州学院, 江苏 泰州 225300; 2. Taizhou University, Taizhou 225300, China

**Keywords:** 超高效液相色谱-串联质谱, 分散固相萃取, 阳离子型金属有机骨架材料, 混合基质膜, 苯氧羧酸类除草剂, 环境水体, ultra performance liquid chromatography-tandem mass spectrometry (UPLC-MS/MS), dispersive solid phase extraction (DSPE), cationic metal-organic frameworks, mixed matrix membrane (MMM), phenoxy acid herbicides, environmental water

## Abstract

离子型金属有机骨架材料(iMOFs)对离子型化合物具有良好的选择吸附性,利用水热法合成了一种金属有机骨架材料MIL-101-NH_2_,以聚偏二氟乙烯(PVDF)为交联剂将其制备成混合基质膜(MMM),然后用三氟甲烷磺酸甲酯进行季胺基功能化改性,最终得到阳离子型金属有机骨架膜材料MIL-101-$NMe_{3}^{+}$-PVDF MMM,采用分散固相萃取方式富集水中的苯氧羧酸类除草剂,建立了一种基于阳离子型MOF混合基质膜的分散固相萃取-超高效液相色谱-串联质谱(UPLC-MS/MS)测定水体中7种苯氧羧酸类除草剂的分析方法。通过傅里叶变换红外光谱和扫描电子显微镜对制备的混合基质膜进行表征,结果表明季胺基功能化改性是成功的,得到了阳离子型MOF膜。对影响萃取效果的主要因素(吸附剂用量、水样pH值、萃取时间、洗脱剂种类、洗脱剂体积及洗脱时间)进行了优化,确定了最佳萃取条件。以0.01%(v/v)甲酸水溶液和乙腈作为流动相进行梯度洗脱,目标化合物在ACQUITY UPLC BEH C18色谱柱(100 mm×2.1 mm, 1.7 μm)上分离,在电喷雾电离源、负离子模式下进行多反应监测(MRM),外标法定量。结果表明,7种苯氧羧酸类除草剂在各自范围内线性关系良好,线性相关系数均大于0.997,方法的检出限(LOD)和定量限(LOQ)分别为0.00010~0.00090 μg/L和0.00033~0.00300 μg/L。在0.005、0.05和0.2 μg/L 3个加标水平下进行加标回收率试验,7种待测物的平均回收率为80%~102%,日内、日间相对标准偏差分别为1.4%~9.4%和4.2%~12.6%。该方法操作简单、快速,灵敏度高,适用于环境水体中7种苯氧羧酸类除草剂的检测。

## Abstract (en)

Phenoxy acid herbicides are widely used because of their excellent efficiency and low cost. However, owing to their strong polarity and water solubility, these herbicides do not degrade easily in a water environment and persist for a long time in water bodies. These herbicides readily enter water bodies via surface runoff, infiltration, and other migration routes, thus affecting water quality safety. Therefore, it is of great significance to establish a sensitive and simple method for the quantitative analysis of phenoxy acid herbicides in environmental water. Given the low concentration of such contaminants in environmental water, appropriate detection methods are important. Ultra performance liquid chromatography-tandem mass spectrometry (UPLC-MS/MS) has high sensitivity and accuracy, thus being well suited for the phenoxy acid herbicides analysis. Sample preparation techniques are also important for the extraction and enrichment of contaminants in environmental water. Dispersive solid phase extraction (DSPE) has attracted considerable attention owing to its low cost, ease of operation, and low solvent consumption. In general, the selectivity and efficiency of solid phase extraction are largely dependent on the characteristics of the solid adsorbent materials. Ionic metal-organic frameworks (iMOFs) have excellent ion-exchange properties and show selective absorptivity to ionic compounds. In this work, a metal-organic framework (MOF) MIL-101-NH_2_ was synthesized by a facile hydrothermal method. Then, a cationic MOF mixed matrix membrane (MMM) was fabricated by soaking the MOFs in a polyvinylidene fluoride (PVDF) solution and further functionalization with quaternary amine groups. A method was developed for the determination of seven phenoxy acid herbicides in water by UPLC-MS/MS based on DSPE. The prepared material was characterized by Fourier transform infrared spectroscopy and scanning electron microscopy to determine its functional groups and morphology. The results showed that there were quaternary amine groups in the material, and that the functionalization did not have any obvious effect on the chemical and crystal structures of MIL-101-NH_2_. The prepared MIL-101-$NMe_{3}^{+}$-PVDF MMM was used as an adsorbent for DSPE to enrich the seven phenoxy acid herbicides in water. It is well known that the key factors influencing extraction efficiency are the adsorption and elution conditions. To establish the optimum extraction conditions, the influence of some important factors, including the adsorbent amount, sample pH, extraction time, elution solvent, elution volume, and elution time, was investigated in detail. Gradient elution was carried out with 0.01% formic acid aqueous solution and acetonitrile as the mobile phase. The target analytes were separated on an ACQUITY UPLC BEH C18 column (100 mm×2.1 mm, 1.7 μm), and multiple reaction monitoring (MRM) was conducted in the negative electrospray ionization mode. The external standard method was used for quantitative analysis. The established method was verified in terms of the linear ranges, limits of detection (LODs), limits of quantification (LOQs), recoveries, and precision. Under the optimal conditions, the seven phenoxy acid herbicides showed good linear relationships in their respective concentration ranges, and the correlation coefficients were all higher than 0.997. The LODs and LOQs were 0.00010-0.00090 μg/L and 0.00033-0.00300 μg/L, respectively. The recoveries were tested at three spiked levels of 0.005, 0.05, and 0.2 μg/L. The average recoveries of the seven compounds were in the range of 80% to 102%. The intra-day and inter-day relative standard deviations were within 1.4% to 9.4% and 4.2% to 12.6%, respectively. The established method was applied to the analysis of the phenoxy acid herbicides in tap water and reservoir water. Three levels of spiked samples were adopted to investigate the accuracy of the method. The results demonstrated that our method is applicable to the detection of trace phenoxy acid herbicides in water samples. In summary, this method has the advantages of simplicity, rapidity, and sensitivity, and it is suitable for the detection of the seven phenoxy acid herbicides in environmental water.

在农业生产中,苯氧羧酸类除草剂因成本低、效果好、除草谱广而被大量使用,但其极性大、水溶性强,在水环境中难降解,会长期残留,且极易通过地表径流和渗透等迁移方式进入各类水体中,从而影响水质安全。研究证明,苯氧羧酸类物质对人体具有毒害作用,可能引发人类软组织恶性肿瘤,干扰内分泌系统,对动物的胎盘、脑组织等也有危害^[1]^。我国《生活饮用水卫生标准》对2,4-二氯苯氧乙酸的限值标准规定为30 μg/L,世界卫生组织对饮用水中苯氧羧酸类除草剂的限值规定为2~100 μg/L^[2,3]^。因此,对水中苯氧羧酸类除草剂的检测具有重要意义。

目前对苯氧羧酸类除草剂的检测方法主要有液相色谱法^[4,5]^、液相色谱-质谱法^[6,7]^、气相色谱法^[8]^和气相色谱-质谱法^[9]^。但由于苯氧羧酸类除草剂在水中的含量较低,因此通常需要对水样进行富集浓缩后才可以准确测定。水中苯氧羧酸类除草剂的样品前处理方法主要包括液液萃取法^[10]^、分散液液微萃取法^[11]^、固相萃取法^[12,13]^和固相微萃取法^[14]^等。

金属有机骨架材料(MOFs)是一类由金属或金属簇与有机配体通过配位键合而形成的纳米多孔材料,具有种类多、比表面积大、孔隙率和孔尺寸可调控等优点,在气体存储^[15]^、药物载体^[16]^、传感^[17]^、催化^[18]^等方面都展现出良好的应用前景。MOFs作为吸附剂及样品前处理的富集材料已有报道^[19,20,21]^。将MOFs制备成膜,使其作为分散固相萃取的吸附剂富集环境水样中的有机污染物,具有操作简单、快速等优势^[22,23]^。但目前大多MOFs材料为中性,对离子型化合物富集效率不高。

离子型MOFs(iMOFs)是一类骨架内带有电荷的新型MOFs材料。与目前报道的多数电中性MOFs相比,iMOFs具有优异的离子交换性能,其对离子型化合物具有良好的选择吸附性^[24,25]^。苯氧羧酸类除草剂在水中通常以离子形态存在,因此本文采用聚偏二氟乙烯(PVDF)与MIL-101-NH_2_材料制备了阳离子型MIL-101-$NMe_{3}^{+}$-PVDF混合基质膜(MIL-101-$NMe_{3}^{+}$-PVDF MMM)作为吸附剂,采用分散固相萃取的前处理方法,结合超高效液相色谱-串联质谱法(UPLC-MS/MS)对水中7种苯氧羧酸类除草剂进行检测,方法简单、快速、准确。

## 1 实验部分

### 1.1 仪器与试剂

QTRAP 3500超高效液相色谱-三重四极杆质谱仪(美国AB Sciex公司), Frontier Nicolet iN10红外光谱仪(美国Thermo Fisher公司), S-4800扫描电子显微镜(日本Hitachi公司), JTN200氮吹仪(杭州聚同电子有限公司), Millipore D-24^UV^超纯水机(美国Millipore公司)。

苯氧羧酸类除草剂标准品:2,4-二氯苯氧乙酸(2,4-D,纯度≥97%)、4-(2,4-二氯苯氧)-丁酸(2,4-DB,纯度≥98%)、2-甲基-4-氯苯氧乙酸(MCPA,纯度>98%)、4-氯苯氧乙酸(4-CPA,纯度≥99%)购自上海阿拉丁化学试剂有限公司,2,4,5-三氯苯氧乙酸(2,4,5-T,纯度>97%)、2-(2,4,5-三氯苯氧基)-丙酸(2,4,5-TP,纯度>98%)、4-苯氧丁酸(PB,纯度>99%)购自德国Dr. Ehrenstorfer公司。色谱纯甲醇、乙腈购自德国Merck公司,色谱纯甲酸购自天津科密欧化学试剂有限公司,2-氨基对苯二甲酸、三氟甲烷磺酸甲酯购自上海麦克林生化科技有限公司,九水合硝酸铬、二氯甲烷、*N*,*N*-二甲基甲酰胺(DMF)、丙酮购自国药集团化学试剂有限公司。

### 1.2 材料的制备

按照文献^[26]^报道采用水热法合成MIL-101-NH_2_。将2-氨基对苯二甲酸(0.36 g)、九水合硝酸铬(0.8 g)、氢氧化钠(0.2 g)加入到15 mL超纯水中,超声混匀,然后将混合物转移到溶剂热反应釜中,在150 ℃条件下反应12 h。冷却至室温后,以8000 r/min离心10 min收集粗产物,然后用DMF和乙醇清洗,在真空干燥箱中干燥12 h,得到金属有机骨架材料MIL-101-NH_2_。

将MIL-101-NH_2_ (40 mg)超声分散在4 mL丙酮中,将PVDF(50 mg)溶于1 mL DMF中,并将其加入到上述MIL-101-NH_2_的悬浊液中,超声混匀,然后将混匀后的溶液涂在玻璃板上,置于70 ℃烘箱中加热使溶剂挥发,制得MIL-101-NH_2_-PVDF MMM。将膜浸泡在6 mL二氯甲烷中并向其中滴加50 μL三氟甲烷磺酸甲酯反应12 h,之后更换二氯甲烷去除未反应的三氟甲烷磺酸甲酯。最后将膜泡在0.1 mol/L盐酸溶液12 h,然后用超纯水反复洗涤酸化的膜材料至洗脱液的pH值达到中性,得到阳离子型MIL-101-$NMe_{3}^{+}$-PVDF MMM。

### 1.3 样品前处理

将MIL-101-$NMe_{3}^{+}$-PVDF MMM置于锥形瓶中,加入50 mL水样,在恒温振荡器中振荡萃取30 min。将膜从水样中取出,用2.5 mL 1.5%氨水甲醇溶液洗脱,共洗脱2次,每次15 min。收集的洗脱液在室温下氮吹至近干,用0.5 mL 20%乙腈水溶液复溶,涡旋混匀后用0.22 μm的滤头过滤,然后进行检测。

### 1.4 仪器条件

色谱条件:色谱柱为ACQUITY UPLC BEH C18色谱柱(100 mm×2.1 mm, 1.7 μm);柱温为40 ℃;流动相为(A)0.01%甲酸水溶液和(B)乙腈;流速为0.4 mL/min。洗脱梯度程序为0~1 min, 80%A; 1~3 min, 80%A~55%A; 3~4 min, 55%A; 4~8 min, 55%A~50%A; 8~10 min, 50%A~20%A; 10~11 min, 20%A; 11~11.1 min, 20%A~80%A; 11.1~13 min, 80%A。进样量为5 μL。

质谱条件:离子源ESI源,负离子模式;多反应监测模式;离子化温度300 ℃;电源电压-4500 V;气帘气压力2.07×10^5^ Pa;雾化气压力3.45×10^5^ Pa;辅助器压力4.14×10^5^ Pa。7种苯氧羧酸类除草剂的其他质谱参数见表1。

**表1:** 7种苯氧羧酸类除草剂的质谱参数

Analyte	t_R_/min	Precursor ions (m/z)	Product ions (m/z)	Declustering potentials/V	Collision energies/eV
4-Chlorophenoxyacetic acid (4-CPA)	3.48	184.7^*^, 186.7	126.8, 128.9	-50, -49	-20, -20
4-Phenoxybutyric acid (PB)	3.83	178.8^*^	93.0	-54	-24
2,4-Dichlorophenoxyacetic acid (2,4-D)	4.17	219.0^*^, 221.0	161.0, 163.0	-40, -40	-19, -19
2-Methyl-4-chlorophenoxyacetic acid (MCPA)	4.29	199.0^*^, 201.0	141.0, 143.0	-50, -50	-22, -22
2,4,5-Trichlorophenoxyacetic acid (2,4,5-T)	4.94	252.7^*^, 254.9	194.8, 196.7	-45, -44	-20, -22
2,4-Dichlorophenoxybutyric acid (2,4-DB)	5.93	247.0^*^, 249.0	161.0, 163.0	-40, -40	-18, -21
2-(2,4,5-Trichlorophenoxy)propanoic acid (2,4,5-TP)	6.09	266.8, 268.7^*^	195.0, 196.9	-48, -45	-16, -25

* Quantitative ion.

## 2 结果与讨论

### 2.1 材料的表征

采用扫描电镜和红外光谱仪对制备的MIL-101-$NMe_{3}^{+}$-PVDF MMM材料进行表征。图1a为膜材料在扫描电镜下的微观结构,可以看出MOFs材料通过聚合物相互交联在一起,其本身的结构没有发生变化,与文献^[25]^报道一致。图1b为膜材料的横截面扫描图,可以看到其具有类似海绵一样的结构,厚度大约为25 μm。

**图1:**
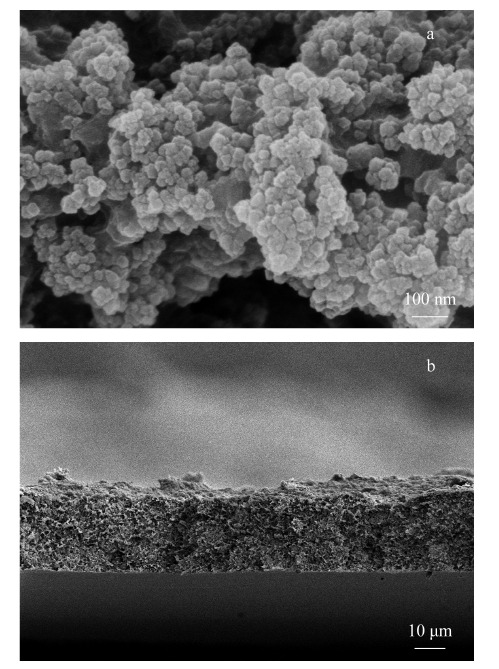
MIL-101-$NMe_{3}^{+}$-PVDF MMM (a)表面和(b)横截面的扫描电镜图

如图2a的PVDF的红外光谱图所示,1173 cm^-1^处的吸收峰与C-F键的伸缩振动有关。如图2b的MIL-101-NH_2_-PVDF MMM红外光谱图所示,3350 cm^-1^和3472 cm^-1^处的吸收峰是由-NH_2_的对称、不对称伸缩振动造成的,1497 cm^-1^和1594 cm^-1^处的峰由苯环中C=C键的伸缩振动引起;而位于1255 cm^-1^和969 cm^-1^处的吸收峰是由于C-N键的伸缩振动,这些结果表明氨基基团存在于MIL-101-NH_2_-PVDF MMM中。由图2c MIL-101-$NMe_{3}^{+}$-PVDF MMM的红外光谱图可以看到,膜改性后C-N键伸缩振动的吸收峰转移到1074 cm^-1^和1280 cm^-1^处,表明了季胺基的存在^[27]^。

**图2:**
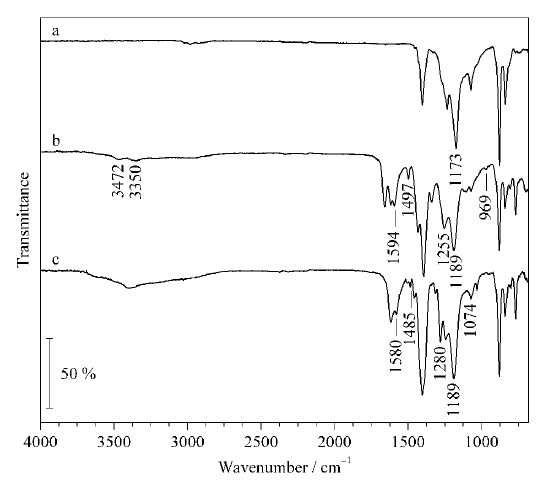
(a)PVDF、(b)MIL-101-NH_2_-PVDF MMM和(c)MIL-101-$NMe_{3}^{+}$-PVDF MMM的红外光谱图

### 2.2 色谱条件的优化

比较了乙腈-水、乙腈-0.01%甲酸水溶液和乙腈-0.05%甲酸水溶液作为流动相时,目标化合物的分离效果和响应情况。结果表明,当流动相中加入甲酸后,7种目标化合物的分离效果明显变好,且响应信号也有所增强,但甲酸浓度过高时会降低化合物的响应信号。因此,最终选择乙腈-0.01%甲酸水溶液作为流动相。7种苯氧羧酸的总离子流色谱图见图3。

**图3:**
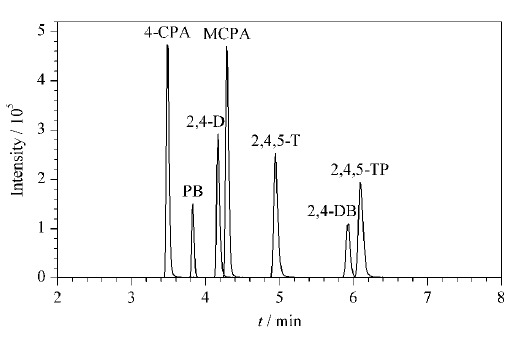
7种苯氧羧酸类除草剂的总离子流色谱图

### 2.3 前处理条件的优化

为了获得最佳萃取效果,考察了影响萃取效率的主要因素:MOF用量、水样pH值、萃取时间、离子强度、洗脱剂类型、洗脱剂体积和洗脱时间。

2.3.1 MOF用量

MOF用量影响吸附活性位点的数量,从而对萃取效果产生较大影响。实验分别考察了MOF用量为5、10、20、40和60 mg时的萃取效果。如图4a所示,当MOF用量从5 mg增加到20 mg, 7种苯氧羧酸类除草剂的萃取效率显著升高,说明随着MOF用量的增加,吸附位点增多,吸附量增加;MOF用量从20 mg增加到40 mg时,回收率趋于平稳;但继续增加MOF用量至60 mg时,7种苯氧羧酸类除草剂的萃取效率则均有下降。因此最终选择MOF用量为40 mg。

**图4:**
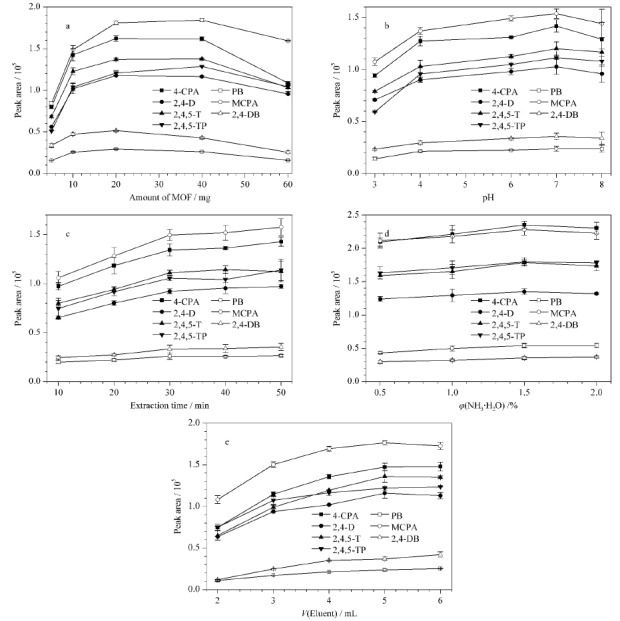
(a) MOF用量、(b)水样pH、(c)萃取时间、(d)洗脱剂氨水浓度和(e)洗脱剂体积对7种苯氧羧酸类除草剂萃取效果的影响(*n*=3)

2.3.2 水样pH值

水样的pH值会影响待测物在水中的存在形式。采用0.1 mol/L盐酸溶液和0.1 mol/L氢氧化钠溶液将水样pH值调至3、4、6、7和8,考察不同pH条件下的萃取效率(见图4b)。结果表明,随着水样pH值的增加,萃取效率逐渐升高,到中性条件下达到最佳,这可能是因为pH值的增加使目标化合物从分子形态变为离子形态,与带正电荷的膜材料之间的静电作用增强,从而增加了萃取效率。pH值继续增加到8后,溶液中的阴离子OH^-^增多,会与目标化合物产生竞争吸附,使萃取效率降低。因此,后续实验保持水样pH值为6~7,对pH在此范围内的自来水或地表水样,可不进行调节。

2.3.3 萃取时间

萃取时间影响目标化合物和材料之间的吸附平衡,从而影响萃取效率。实验考察了萃取时间分别为10、20、30、40和50 min时7种苯氧羧酸类除草剂的萃取效率。如图4c所示,萃取时间由10 min增加到30 min时,萃取效率明显升高,继续延长萃取时间,萃取效率无显著增加,说明30 min吸附基本达到平衡。因此实验选取萃取时间为30 min。

2.3.4 离子强度

通过调节水样中氯化钠的浓度(0、1、5、10和20 mmol/L),考察离子强度对萃取效率的影响。结果表明,随着NaCl浓度的增加,7种苯氧羧酸类除草剂的峰面积均呈现下降趋势,说明盐的加入不利于目标物质的萃取,可能是因为Cl^-^与目标物质之间存在竞争吸附,因此后续实验选择不添加NaCl。

2.3.5 洗脱条件

洗脱剂是影响萃取效率的重要因素。甲醇对苯氧羧酸类除草剂的洗脱效果较好,氨的加入有利于酸性物质的洗脱,因此考察了氨水甲醇溶液中氨水的体积分数(0.5%、1.0%、1.5%和2.0%)对洗脱效果的影响(见图4d)。随着氨水体积分数从0.5%增加到1.5%, 7种苯氧羧酸的峰面积逐渐提高,而氨浓度继续升高后,有3种苯氧羧酸的峰面积降低,所以选择1.5%氨水甲醇作为洗脱剂。

洗脱剂的体积过小会导致目标物质的洗脱不彻底,影响萃取效率;洗脱剂的体积太大则会增加后续浓缩的时间。因此考察了单次洗脱剂用量为1.0、1.5、2.0、2.5和3.0 mL时,不同体积分别洗脱两次条件下的洗脱效果。如图4e所示,总洗脱剂用量从2 mL增加到5 mL, 7种苯氧羧酸类除草剂的峰面积逐渐升高,用量继续增大后,峰面积无显著增加。因此选取单次洗脱剂用量为2.5 mL,洗脱两次。

洗脱时间同样影响萃取效率。实验考察了洗脱3、6、10、15和20 min,连续洗脱两次条件下的洗脱效果。结果表明,单次洗脱时间从3 min增加至15 min,萃取效率没有显著的变化,但总体呈上升趋势,而继续增加洗脱时间至20 min,萃取效率稍有降低。因此,为保证萃取效果,选取单次洗脱时间为15 min,洗脱两次。

### 2.4 方法学验证

2.4.1 线性范围、检出限和定量限

对0.001、0.005、0.01、0.05、0.1、0.2和0.5 μg/L的系列不同浓度的模拟水样检测,以质量浓度为横坐标、峰面积为纵坐标绘制标准曲线。结果如表2所示,7种苯氧羧酸类除草剂在各自范围内具有良好的线性关系,相关系数(*r*^2^)均大于0.997,检出限(LOD, *S/N*=3)和定量限(LOQ, *S/N*=10)分别为0.00010~0.00090 μg/L和0.00033~0.00300 μg/L。

**表2:** 7种苯氧羧酸类除草剂的线性方程、相关系数、线性范围、检出限和定量限

Analyte	Linear equation	r^2^	Linear range/(μg/L)	LOD/(μg/L)	LOQ/(μg/L)
4-CPA	y=9.63×10^2^x+2.29×10^3^	0.9979	0.001-0.5	0.00013	0.00042
PB	y=1.85×10^2^x+9.04×10^2^	0.9981	0.005-0.5	0.00045	0.00150
2,4-D	y=7.74×10^2^x+2.69×10^3^	0.9998	0.001-0.5	0.00013	0.00042
MCPA	y=1.20×10^3^x+1.44×10^3^	0.9995	0.001-0.5	0.00010	0.00033
2,4,5-T	y=9.02×10^2^x+2.87×10^3^	0.9992	0.001-0.5	0.00015	0.00050
2,4-DB	y=2.77×10^2^x+4.63×10^2^	0.9999	0.005-0.5	0.00090	0.00300
2,4,5-TP	y=7.81×10^2^x-5.87×10^2^	0.9978	0.001-0.5	0.00015	0.00050

*y*: peak area; *x*: mass concentration, μg/L.

2.4.2 回收率和精密度

在空白水样中添加不同体积的标准溶液,配制成苯氧羧酸类除草剂质量浓度分别为0.005、0.05、0.2 μg/L的加标水样,进行加标回收试验。每个浓度点1 d内测定5个平行样,考察日内精密度,连续测定5 d,考察日间精密度。结果如表3所示,7种苯氧羧酸类除草剂的加标回收率为80%~102%,日内和日间精密度分别为1.4%~9.4%和4.2%~12.6%。

**表3:** 7种苯氧羧酸类除草剂的回收率和精密度(*n*=5)

Analyte	Spiked/ (μg/L)	Recovery/ %	RSDs/%		Analyte	Spiked/ (μg/L)	Recovery/ %	RSDs/%	
			Intra-day	Inter-day				Intra-day	Inter-day
4-CPA	0.005	80	5.7	4.2	2,4,5-T	0.005	88	4.7	6.0
	0.05	89	2.4	7.3		0.05	92	2.9	10.7
	0.2	92	1.4	5.6		0.2	89	4.7	6.0
PB	0.005	81	8.8	7.2	2,4-DB`	0.005	81	8.9	12.3
	0.05	93	4.4	6.0		0.05	85	7.9	12.6
	0.2	91	3.9	4.9		0.2	82	8.0	9.8
2,4-D	0.005	81	7.4	9.8	2,4,5-TP	0.005	102	9.4	10.8
	0.05	91	3.9	5.2		0.05	91	3.2	8.2
	0.2	89	3.1	4.6		0.2	88	3.1	8.2
MCPA	0.005	91	7.0	5.2					
	0.05	92	3.0	7.3					
	0.2	84	2.8	5.1					

### 2.5 实际样品分析

利用建立的方法对实验室自来水和水库水进行分析检测,验证该方法在实际应用中的可行性。结果如表4所示,自来水中没有检测到苯氧羧酸类除草剂的存在;在水库水中检测到微量的2,4,5-TP,含量为0.004 μg/L,远低于世界卫生组织对饮用水中该物质的限值规定(9 μg/L)。

**表4:** 实际水样中7种苯氧羧酸类除草剂的分析结果(*n*=3)

Analyte	Spiked/(μg/L)	Tap water				Reservoir water		
		Found/(μg/L)	Recovery/%	RSD/%		Found/(μg/L)	Recovery/%	RSD/%
4-CPA	0	ND				ND		
	0.01	0.008	80	3.5		0.007	72	4.1
	0.05	0.043	86	0.6		0.046	92	2.6
	0.2	0.178	89	8.3		0.171	86	4.9
PB	0	ND				ND		
	0.01	0.009	90	5.5		0.008	80	2.4
	0.05	0.044	88	2.3		0.045	90	4.1
	0.2	0.182	91	2.5		0.179	90	6.5
2,4-D	0	ND				ND		
	0.01	0.008	80	11.3		0.009	90	7.6
	0.05	0.042	84	3.1		0.047	94	2.9
	0.2	0.179	90	2.3		0.168	84	1.5
MCPA	0	ND				ND		
	0.01	0.008	80	9.5		0.009	90	5.4
	0.05	0.043	86	2.8		0.043	86	3.3
	0.2	0.167	84	4.4		0.158	79	4.2
2,4,5-T	0	ND				ND		
	0.01	0.008	80	8.1		0.008	80	11.5
	0.05	0.046	92	2.4		0.045	90	3.2
	0.2	0.175	88	4.6		0.178	89	5.1
2,4-DB`	0	ND				ND		
	0.01	0.010	102	6.2		0.011	110	7.5
	0.05	0.045	90	6.4		0.063	126	10.4
	0.2	0.166	83	9.6		0.214	107	6.6
2,4,5-TP	0	ND				0.004		
	0.01	0.009	90	0.7		0.013	90	11.0
	0.05	0.047	94	8.1		0.047	86	9.1
	0.2	0.176	88	3.3		0.153	75	6.0

ND: not detected.

### 2.6 与文献方法比较

为了比较所建方法的分析性能,比较了文献报道的水体中苯氧羧酸类除草剂的分析方法(见表5)。与文献方法相比,本方法采用分散固相萃取,通过将吸附剂制备成膜的形式,使材料更容易与水分离,不需要离心、过滤等步骤,操作简单,且对于pH值在6~7附近的水样可以不调节pH直接分析,节省了前处理时间;同时,本方法的检出限也相对较低。

**表5:** 本方法与文献报道的苯氧羧酸类除草剂分析方法比较

Material	Method	Number of analytes	Matrices	Practicable pH	LOD/(μg/L)	Ref.
NH_2_-MWCNTs	MSPE-UPLC-MS/MS	7	lake, river, farmland and tap water	5.4	0.01000-0.02000	[12]
PP-CMPs	DSPE-LC-MS/MS	5	tap, spring and pond water	3	0.00055-0.00384	[13]
UiO-66-NM-PVDF MMM	DME-UPLC-MS/MS	6	sewage and reservoir water	3-6	0.00003-0.00059	[24]
V-g-C_3_N_4_	DSPE-DART-MS	3	tap and lake water	2	0.00050-0.00200	[28]
MIL-101	DSPE-UPLC-MS/MS	12	river water	6	0.00018-0.00088	[29]
MIL-101-NM-PVDF MMM	DSPE-UPLC-MS/MS	7	tap and reservoir water	6-7	0.00010-0.00090	this work

MWCNTs: multi-walled carbon nanotubes; PP-CMPs: polyphenylene-based conjugated microporous polymers; V-g-C_3_N_4_: velvet-like graphitic carbon nitride; MSPE: magnetic solid phase extraction; DSPE: dispersive solid phase extraction; DME: dispersive membrane extraction.

## 3 结论

苯氧羧酸类除草剂在水中容易电离,通常以阴离子形式存在。本研究以MIL-101-$NMe_{3}^{+}$-PVDF MMM作为分散固相萃取吸附剂,通过阴阳离子间的静电相互作用,实现对7种苯氧羧酸类除草剂的吸附,结合UPLC-MS/MS检测,建立了同时检测水体中7种苯氧羧酸类除草剂的分析方法。该方法灵敏度、准确度满足实际样品检测要求,具有较好的实际应用前景。
